# Standardized Three-Dimensional Lateral Distraction Test: Its Reliability to Assess Medial Canthal Tendon Laxity

**DOI:** 10.1007/s00266-021-02440-y

**Published:** 2021-07-07

**Authors:** Xiaoyi Hou, Alexander C. Rokohl, Marius M. Meinke, Jinhua Liu, Senmao Li, Wanlin Fan, Ming Lin, Renbing Jia, Yongwei Guo, Ludwig M. Heindl

**Affiliations:** 1 Department of Ophthalmology, West China Hospital, Sichuan University, Chengdu, China; 2 Department of Ophthalmology, Faculty of Medicine and University Hospital Cologne, University of Cologne, Kerpener Strasse 62, 50937 Cologne, Germany; 3 Department of Ophthalmology, Xi’an Fourth Hospital, Xi’an, Shaanxi China; 4 Department of Ophthalmology, Ninth People’s Hospital, Shanghai Jiao Tong University School of Medicine, Shanghai, China; 5 Eye Center, Second Affiliated Hospital, School of Medicine, Zhejiang University, 88 Jiefang Road, Hangzhou, 310009 China; 6 Center for Integrated Oncology (CIO) Aachen-Bonn-Cologne-Duesseldorf, Cologne, Germany

**Keywords:** Medial canthal tendon laxity, Lateral distraction test, Three dimensional stereophotogrammetry, Reliability, Eyelid

## Abstract

**Background:**

Assessment of MCT laxity is critical to the surgery options. Our study aimed to analyze the reliability of measuring medial canthal tendon (MCT) laxity by using a novel standardized three-dimensional lateral distraction test (3D-LDT).

**Methods:**

Forty-eight Caucasian volunteers (25 males and 23 females, 96 eyes) between 22 and 84 years of age (55.6 ± 18.6 years old) were included in our study. From a neutral position, the lower eyelid was gently pulled laterally along a horizontal line to define the most distracted position of the lower punctum. Both in the neutral and distracted position, standardized 3D images were acquired for each subject by two observers, and each image were measured twice by two raters. Four landmarks and six corresponding linear measurements were evaluated for intra-rater, inter-rater, and inter-method reliability.

**Results:**

Intra-rater, inter-rater and inter-method reliability analyses of 3D-LDT revealed an intraclass correlation of more than 95%, a mean absolute difference of less than 1 mm, and a technical error of measurement of less than 1 mm. Measurements of relative error (2.59–12.04%) and relative technical error (1.83–16.05%) for the inter-landmarks distance from pupil center to the lower punctum were higher than those from limbus nasal center to the lower punctum (6.13–30.39 and 4.34–26.85%, respectively).

**Conclusions:**

This study provided high reliability of the three-dimensional lateral distraction test (3D-LDT) for assessing medial canthal tendon (MCT) laxity, which were never evaluated by digital imaging system.

**Level of Evidence IV:**

This journal requires that authors assign a level of evidence to each article. For a full description of these Evidence-Based Medicine ratings, please refer to the Table of Contents or the online Instructions to Authors www.springer.com/00266.

## Introduction

The medial canthal tendon (MCT) is a stripe of fibrous tissue that insert into the orbicularis oculi muscle as a ligament [1]. Aging may cause laxity of MCT, which eventually contribute to the etiology of lower eyelid ectropion, specifically medial ectropion and the accompanying epiphora [2, 3]. Proper assessment of MCT laxity is critical to the surgery options. A deficient diagnosis of neglecting the laxity [4] and thereby incomplete correction ectropion may lead to recurrent of epiphora and redness of the inferonasal conjunctiva as a result [5].

Lateral distraction test (LDT) was the gold traditional method for assessing the laxity of MCT, which was performed by pulling the lower eyelid laterally along a horizontal direction and observing how far the lower punctum can be pulled in relation to the cornea nasal limbus [6, 7] Results may vary depending on the subject of the issue, and quantitative analysis is difficult. Several studies [4, 6–12] have investigated the grading method of MCT laxity. However, no universally grading scale or format is accepted at present for recording the laxity, and the result is usually just noted as being present or not.

Noninvasive three-dimensional (3D) digital photogrammetry has recently gained much interest in anthropometry and is beginning to replace the classical anthropometric techniques, including the caliper and two-dimensional (2D) imaging measurements [13]. Several 3D digital imaging systems have been developed and already used successfully in craniofacial centers all over the world [14–20]. In addition to linear distances and angles calculation, 3D imaging system dramatically offers calculation of surface curves, surface areas, volumes and volume change from the human surface. Recently, the reliability and accuracy of applying VECTRA^M3^ (Canfield Scientific, Inc., Parsippany, NJ), a type of 3D stereo-photography system, in maxillofacial anthropometry has been validated in several studies [14, 21–26]. More specifically, the feasibility and reliability of employing this 3D imaging system in the periocular region of this 3D imaging system has also been verified by previous studies [13, 27, 28].

As far as we know, the MCT laxity has never been quantified by using 3D imaging system, and there is an increasing need for developing an appropriate way to quantify it. Hence, based on the successful application of this 3D imaging technique, we developed a novel 3D lateral distraction test (3D-LDT) to modify the assessment of MCT laxity. In addition, we want to investigate the reliability and reproductivity of this 3D-LDT, so as to utilize this new technique in the clinical routine for assessing the laxity of MCT. Therefore, the purposes of this study were to investigate the reliability of 3D camera when operating the LDT, and to assess the interobserver reproducibility of LDT using 3D stereophotogrammetry.

## Material and Methods

### Subjects

Forty-eight Caucasian volunteers (25 males and 23 females, 96 eyes) between 22 and 84 years of age (55.6 ± 18.6 years old) were included in our study. Each participant had normal eyelids, no history of previous eyelid or ocular surgery, no history of ocular disease or long term of eye drops application. Individuals with facial pathologies, malformations, severe asymmetry, or medical histories of injuries modifying the periocular morphology were excluded. Subjects were randomly recruited from the Department of Ophthalmology, University Hospital of Cologne, Germany. The study protocol adhered to the Declaration of Helsinki and was approved by the Institutional Review Board of the University of Cologne.

### Data Collection

VECTRA^M3^ 3D Imaging System produced by Canfield Scientific, Inc., Parsippany, NJ, was applied for capturing the data of the observed periocular surface [27]. Calibration [13] of the VECTRA system was performed daily, prior to patient arrival, or whenever the system has been moved or altered. Following calibration, each participant was positioned according to the manufacturer’s instructions. A skilled operator performed all the captures according to the manufacturer’s instructions in standard environment under the same ambient lighting circumstance. All the 3D facial models were elaborated by the VECTRA Analysis Module (VAM) software for surface topography measuring, analyzing, and manipulating.

### Neutral Position

**Fig. 1:**
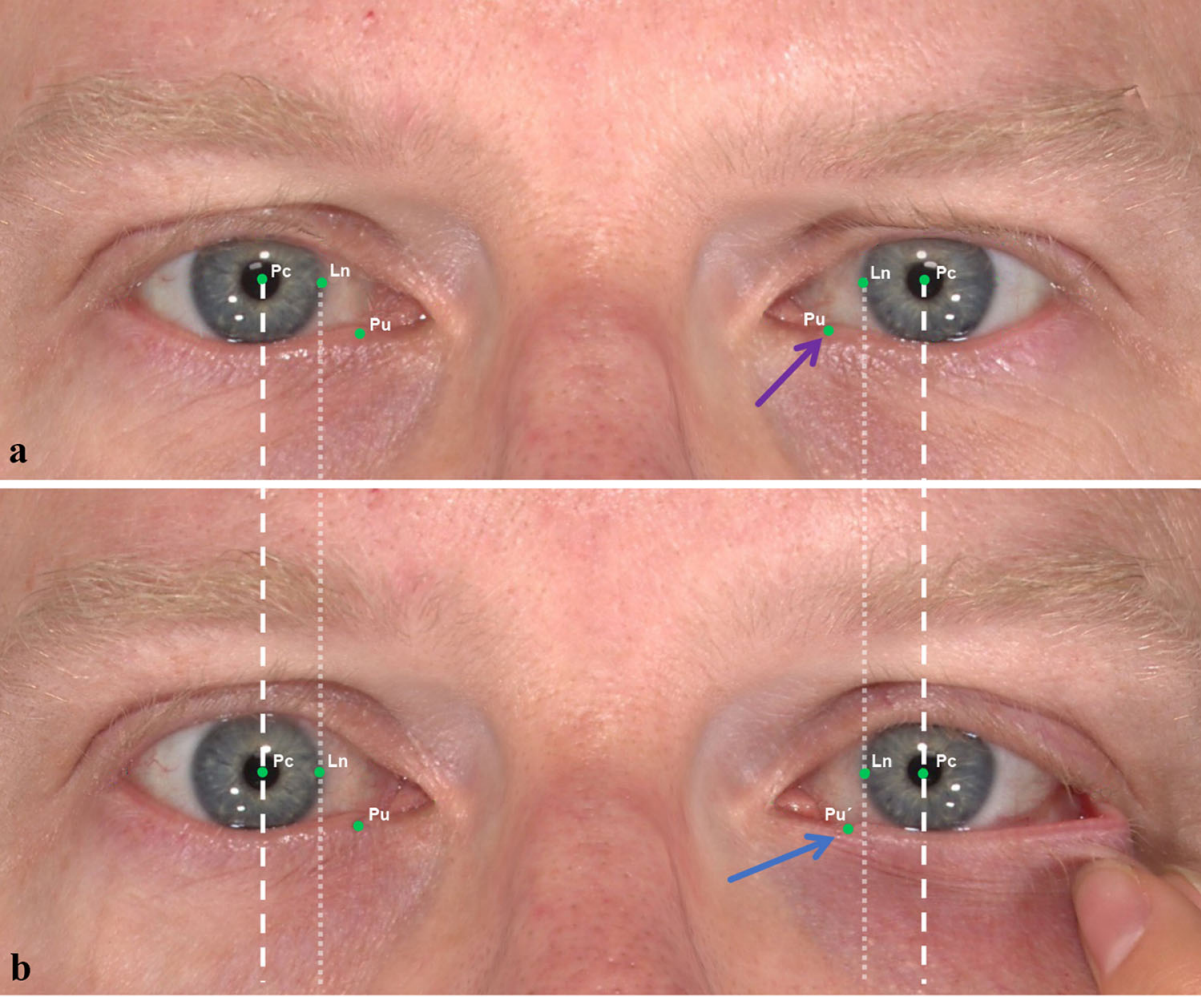
Three-dimensional Neutral Position (NP) and Lateral Distraction Test (LDT) for a 32-year-old male participant. **a** NP images were taken without any expression and any distraction test of the lower eyelid. Three landmarks (Pc, Ln, Pu) were included. Linear distances were based on these landmarks. Dotted lines are vertical lines across medial corneoscleral limbus and dashed lines are vertical lines across pupil center. **b** LDT images were taken when performing the distraction test of the lower eyelid mentioned above. Three landmarks (Pc, Ln, Pu´) on the tested eye and 3 landmarks (Pc, Ln, Pu) on the opposite eye were included in our research. Pu (purple arrow) represented for the marked resting punctum and Pu´ represented for the distracted punctum (blue arrow)

For each volunteer, the former 3D images were taken without any expression and any distraction test of the lower eyelid mentioned above (Neutral position, NP). Before image acquisition, we marked the lower punctum of both eyelids with a black pen for further analyzes. During the acquisition, each participant was asked to keep the eyes simultaneously opening and to look forward into the mirror hanging in the upper middle of the 3D camera. Then, the operator pressed the bottom and got the image for the NP (Fig. 1a).

### Distracted Position: Lateral Distraction Test (LDT)

Lateral distraction test (LDT) was the golden method for assessing the MCT [8]. The examiner gently pulled the lateral part of the lower eyelid along a horizontal direction until it can no longer be pulled to the lateral any further. Each participant was placed the same way as NP in front of the VECTRA^M3^ camera, while the 3D images were taken when operating the LDT (Fig. 1b).

### Landmarks and Linear Distances

**Fig. 2:**
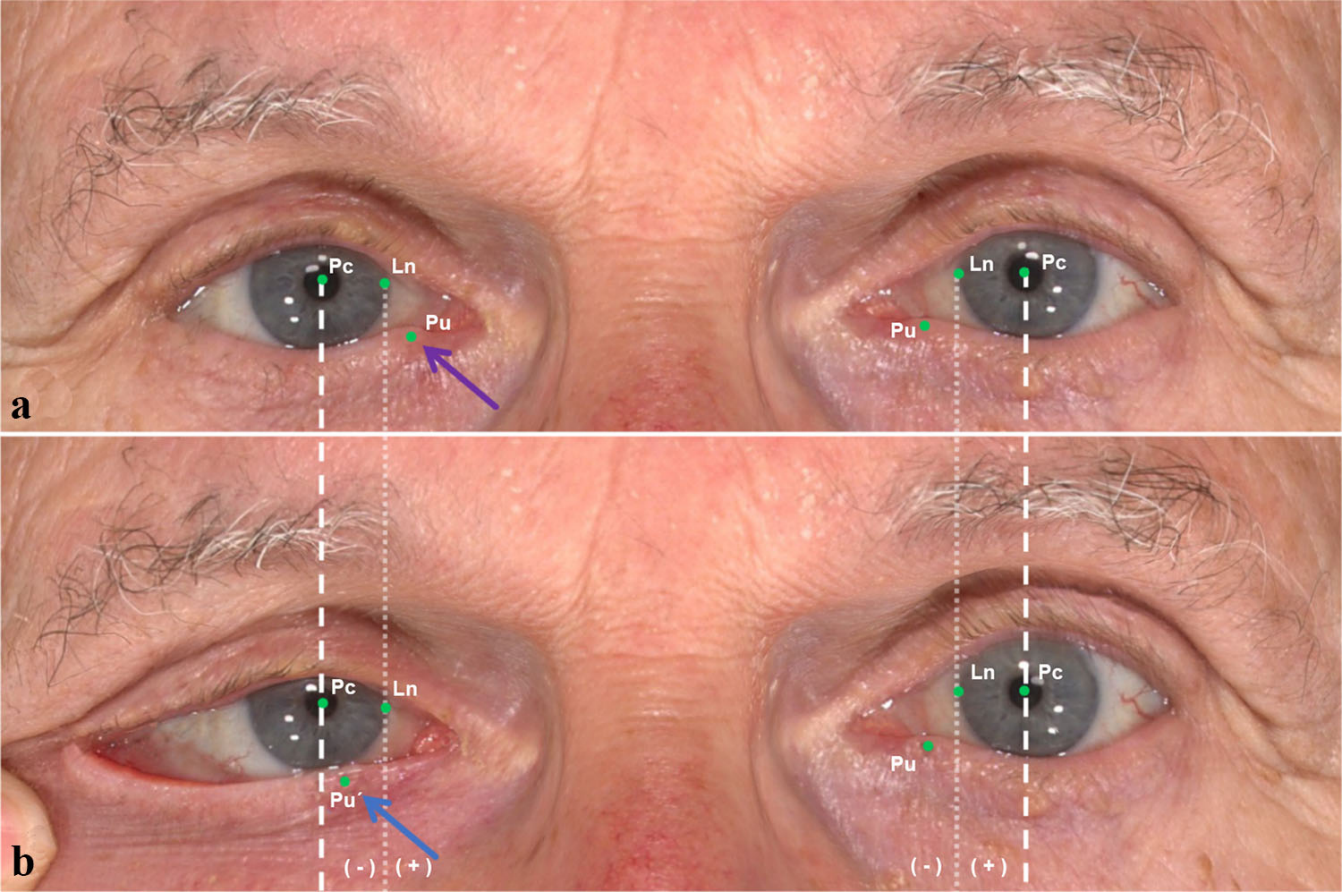
Three-dimensional Neutral Position (NP) and Lateral Distraction Test (LDT) for an 80-year-old male participant. **a** NP images were taken without any facial expression. **b** LDT images were taken when performing the distraction test of the lower eyelid. The position is recorded with ´negative (−) ´, if the pu´ is lateralized to the vertical line through the medial corneoscleral limbus. The position is recorded with ´positive (+) ´, if the pu´ is medialized to the vertical line through the medial corneoscleral limbus. The figure shows a positive (+) location of the (pu´) for this participant (blue arrow)

Three basic landmarks were used in each image, including pupillary center (Pc), punctum (Pu) and the medial corneoscleral limbus point (Ln). In the neutral position (NP), the fourth landmark was the intersection of the horizontal line through the pupil center and the vertical line through the neutral punctum (Pu). The linear distance from Pu to the vertical line across Pc was recorded nPc-Pu and the linear distance from Pu to vertical line across Pc recorded nLn-Pu in NP, which were the basis for the following measurements (Fig. 2a). In the lateral distraction test (LDT), the fourth landmark was the intersection of the horizontal line through the pupil center and the vertical line through the distracted punctum (Pu´). The linear distance from Pu´ to the vertical line across Pc was recorded dPc-Pu´ and the linear distance from Pu´ to vertical line across Ln recorded dLn-Pu´ in LDT (Fig. 2b). The linear distance from Pu´ to the vertical line across Pc was recorded n*Pc-Pu and the linear distance from Pu to vertical line across Ln recorded n*Ln-Pu´ in LDT for the opposite eyes (Table 1).

**Table 1:** Definition of landmarks

Landmarks	Definition
Pc	Pupillary center
Ln	Medial corneoscleral limbus point
Pu	The mark on the skin representing the position of lower punctum
Pu´	The distracted position of the lower punctum
nPc-Pu	Horizontal distance from pupil center (Pc) to lower punctum (Pu) in NP
nLn-Pu	Horizontal distance from medial corneoscleral limbus point (Ln) to lower punctum (Pu) in NP
dPc-Pu´	Horizontal distance from pupil center (Pc) to lower punctum (Pu´) in LDT
dLn-Pu´	Horizontal distance from limbus nasal center (Ln) to lower punctum (Pu´) in LDT
n*Pc-Pu	Horizontal distance from pupil center (Pc) to lower punctum (Pu´) of the opposite eye in LDT
n*Ln-Pu	Horizontal distance from medial corneoscleral limbus point (Ln) to lower punctum (Pu´) of the opposite eye in LDT

*n* normal distance; n* normal distance in LDT; *d* distraction distance; *NP* neutral position; *LDT* lateral distraction test.

### Intra- and Inter-Rater Reliability of Lateral Distraction Test by Using VECTRA^M3^ System

To avoid the bias of the distraction test caused by imprecision [14] and inaccuracy [13], we analyzed the intra- and inter-rater precision and accuracy of lateral distraction test by using VECTRA^M3^ 3D system. For intra-rater reliability, initial inter-landmark measurements were acquired on 3D images and calculated by the first rater (X. Hou) using the VAM software. These measurements were repeatedly calculated at least two weeks later by the same observer on the same images. For inter-rater reliability, both initial inter-landmark measurements on the same images were acquired by the first rater (X. Hou) and the second rater (A.C. Rokohl).

### Inter-Methods Reliability of Lateral Distraction Test by Using VECTRA^M3^ System

Comparison between neutral and distracted position plays a vital role as possible error might occur when performing different captures in different times or by different observers. Hence, we performed two sets of comparison.

The first comparison was performed in the images of NP. Each subject got two captures by the same observer (X.H.) at an interval time of about 45 min, and a new calibration was performed in the gap. Then, landmarks and inter-landmarks measurements for each capture were performed by the same observer (X.H.) after 24 hours.

Secondly, LDT of the same participant was performed by two examiners X.H. and the second observer (A.C. R.) at an interval of approximately 45 min. Then, both observers (X.H. and A.C.R.) put the landmarks and performed the measurements on their own-operated images of the same participant using VAM software. Both observers (X.H. and A.C.R.) were masked to the others’ measurements and very experienced in using VECTRA^M3^ 3D system.

### Potential Clinical Indications

**Fig. 3:**
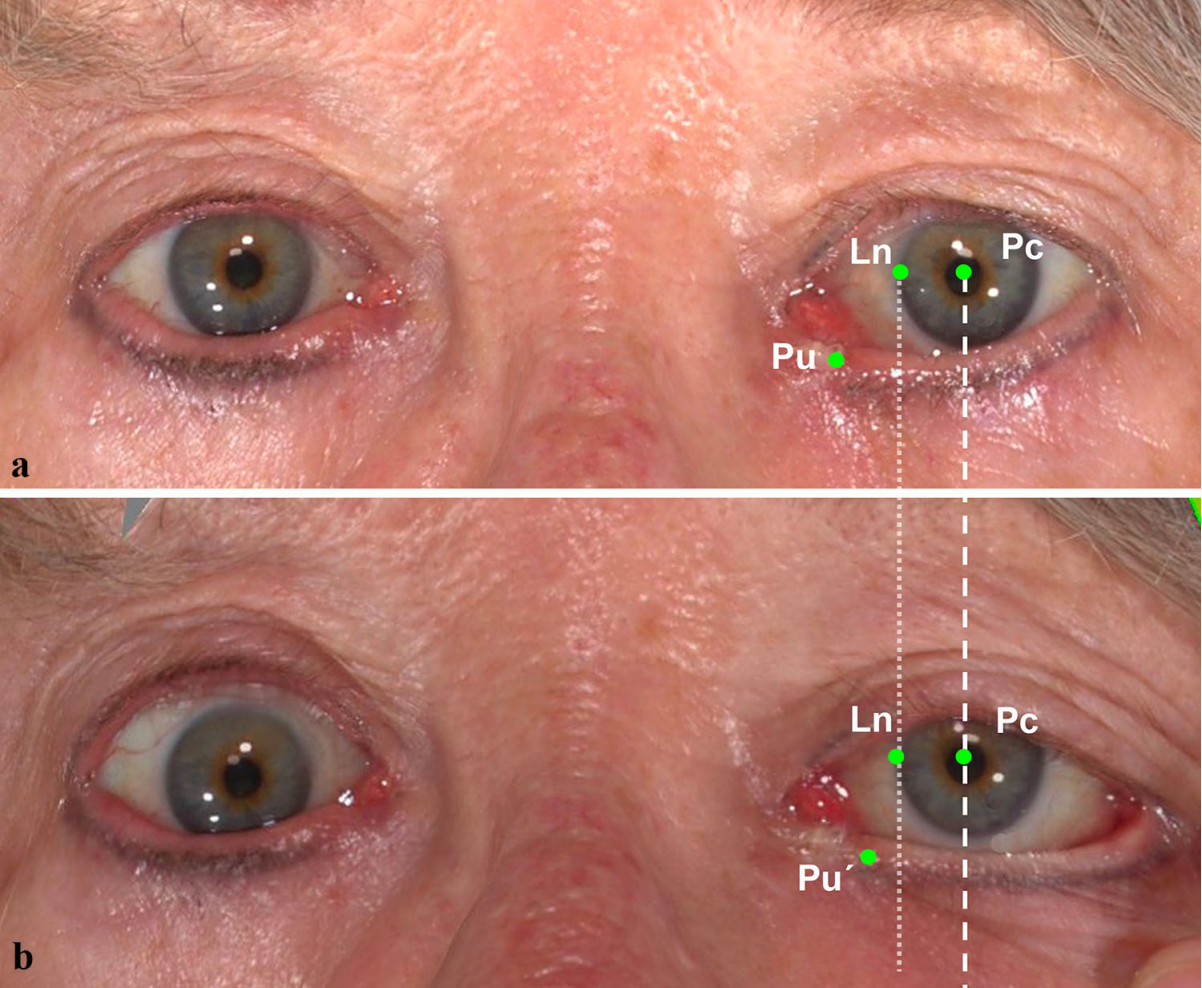
Three-dimensional photographs in neutral position (**a**) and performing the lateral distraction test (**b**) were taken in a 72-year-old female participant before correction surgery of medial ectropion (left eye), which was secondary to a Basel cell carcinoma excision 6 months ago. An obvious medial ectropion was shown in the NP and the punctum displacement was calculated as 2.12 mm by our landmark system

**Fig. 4:**
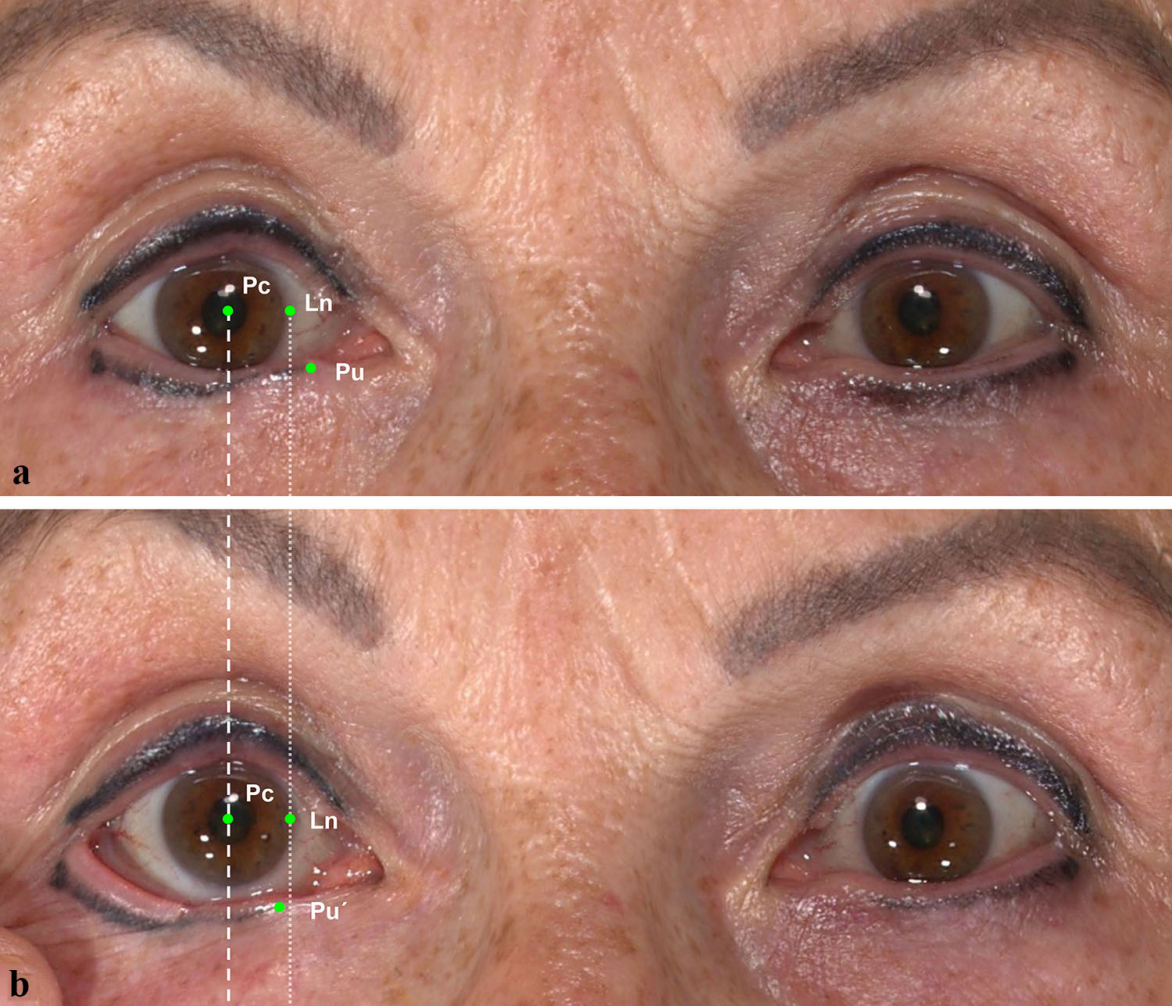
Three-dimensional photographs in neutral position (**a**) and performing lateral distraction test (**b**) were taken in a 61-year-old female participant who underwent Hughes plastic surgery following basal cell carcinoma excision (right eye) three years ago. A good post-operative position was shown in the NP and the LDT displacement was calculated as 2.96 mm by our landmark system

Both 3D-NP and 3D-LDT were performed on patients with different clinical status including post-operative images for pre-operative images for patients with involutional ectropion (Fig. 3), and patients with basal cell carcinoma who underwent Huges plastic surgery (Fig. 4).

## Statistical Analyses

Each participant got four pictures. The former two pictures of NP were performed by the first author (X.H.), the latter two pictures of LDT were performed by the first author and the second observer (A.C.R.), separately. We calculated five statistics (Table 2) to evaluate inter-rater, intra-rater and inter-method reliabilities [29, 30]. Inter-rater and intra-rater reliability were performed on the same images. Inter-method reliability included comparing two neutral captures by one observer and comparing LDT performed by X.H. and A.C.R. The intraclass correlation coefficient (ICC) demonstrates high reliable result when close to 1 and low reliable result when close to 0 [18]. Mean absolute difference (MAD) was computed by equally dividing the absolute difference between two measurements. The relative error measurement (REM) was computed by averaging the MAD using the grand mean of two measurements for a specific variable and multiplying the result by 100 [13]. The technical error of measurement (TEM) was calculated as $$\left( {\sqrt {{\raise0.7ex\hbox{${\left( {\sum D^{2} } \right)}$} \!\mathord{\left/ {\vphantom {{\left( {\sum D^{2} } \right)} {2N}}}\right.\kern-\nulldelimiterspace} \!\lower0.7ex\hbox{${2N}$}}} } \right)$$, in which D is the difference between independent measurements and N is the count of measured subjects [14]. This statistic can be explicated similar to the standard deviation [15]. The relative TEM (%TEM or rTEM) has been introduced to compare imprecision of different variables due to the positive association between the size of measurement and TEM. It was also computed by averaging the TEM for a specific variable by its grand mean and multiplying by 100. According to the scale initiated by Andrade et al., these results were defined into five categories: less than 1%, excellent; 1–3.9%, very good; 4–6.9%, good; 7–9.9%, moderate; and more than 10% poor [31, 32].

**Table 2:** Summary of reliability estimates evaluated

Statistic	Equation
Intraclass correlation coefficient (ICC)	$B/B + W$
Mean absolute difference (MAD)	$|X_{1} - ~X_{2} |$
Technical error of measurement (TEM)	$$\sqrt {{\raise0.7ex\hbox{${\left( {\sum D^{2} } \right)}$} \!\mathord{\left/ {\vphantom {{\left( {\sum D^{2} } \right)} {2N}}}\right.\kern-\nulldelimiterspace} \!\lower0.7ex\hbox{${2N}$}}}$$
Relative error measurement (REM)	$MAD/X_{3} \times 100$
Relative TEM (% TEM or rTEM)	$TEM/X_{3} \times 100$

*B* between-measurement variance; *W* within measurement variance; [7] *D* difference between measurements; *N* number of eyes or subjects measured; *X1* mean for rater 1 (session 1, or session 2 of capture 1); *X2* mean for rater 2 (session 2, or session 2 of capture 2); *X3* grand mean.

Microsoft Excel 2016 for MAC (Microsoft Corp., Redmond, WA, USA) was applied to record all the original data of inter-landmarks measurements. Subsequently, Statistical analysis was performed by SPSS version 23 software (IBM Corp., Armonk, NY, USA). Intra-rater, inter-rater, and inter-method differences of all the measurements were calculated using this system. Paired-sample *t* test was performed for normally distributed data and Wilcoxon signed rank tests for paired data were applied for non-normally distributed data. *P* values ≤ 0.05 were considered statistically significant.

## Results

Demographic features of the study subjects are shown in Table 3. A total of 48 participants was included in our study. Study participants were between 22 and 84 years of age (55.6 + 18.6 years old). The ratio of men to women was nearly equal, and most study participants were white, non-Hispanic. Results of reliability were divided into three categories including intra-rater, inter-rater and inter-method reliability. Table 4 demonstrated the ICC and mean differences of intra-rater, inter-rater, and intra-method for all inter-landmark measurements. Estimates of MAD, TEM, REM, and %TEM is shown in Table 5. Although statistically significant differences were found between intra-rater, inter-rater, or inter-method measurements, the different magnitudes for all of them were less than 1 mm and not clinically significant.

**Table 3:** Demographic characteristics of study participants

Categories	Count
*Age*	
Range (years old)	22–84 yrs
Mean ± SD	55.6 ± 18.6 yrs
*Sex*	
Male	25 (52.1%)
Female	23 (46.9%)
Total	48
*Race/ethnicity*	
White/non-hispanic	48 (100.0%)
Other	0

**Table 4:** Intraclass correlation coefficient (ICC) and mean differences (Dmean) of intra-rater, inter-rater inter-methods across all measurements on three-dimensional images

Landmarks		Intra-rater			Inter-rater			Inter-methods		
		ICC(CI95%)	D- mean	*P* value	ICC(CI95%)	D-mean	*P* value	ICC(CI95%)	D- mean	*P* value
NP	nPc-Pu	0.98 (0.96–0.99)	0.05	0.41	0.97 (0.94–0.98)	0.20	0.001	0.94 (0.68–0.98)	0.48	$<0.001$
	nLn-Pu	0.98 (0.96–0.99)	0.08	0.18	0.98 (0.96–0.99)	− 0.07	0.27	0.87(0.77–0.93)	0.15	0.29
LDT	dPc-Pu´	0.98 (0.96–0.99)	0.11	0.14	0.95 (0.90–0.97)	− 0.11	0.35	0.77 (0.59–0.87)	0.14	0.52
	dLn-Pu´	0.98 (0.96–0.99)	0.08	0.36	0.97 (0.94–0.98)	− 0.22	0.33	0.78 (0.59–0.88)	− 0.01	0.98
	n*Pc-Pu	0.98 (0.97–0.99)	0.06	0.28	0.98 (0.95–0.99)	− 0.34	$<0.001$	0.78 (0.60–0.88)	0.09	0.59
	n*Ln-Pu	0.95 (0.92–0.97)	0.17	0.07	0.93 (0.88–0.96)	0.04	0.74	0.65 (0.36–0.81)	0.42	0.06

*NP* Neutral Position; *LDT* Lateral Distraction Test

**Table 5:** Intra-rater, inter-rater reliability and inter-methods reliability of all measurements on 3D images

Landmarks		Intra-rater				Inter-rater				Inter-methods			
		MAD	RED	TED	%TEM	MAD	RED	TED	%TEM	MAD	REM	TEM	%TEM
NP	nPc-Pu	0.26	2.76	0.30	3.17	0.36	3.82	0.28	2.91	0.24	2.59	0.17	1.83
	nLn-Pu	0.27	6.99	0.31	8.15	0.34	9.00	0.26	6.76	0.24	6.13	0.17	4.34
LDT	dPc-Pu	0.41	6.21	0.39	6.00	0.50	7.5	0.62	9.52	0.83	12.04	1.11	16.05
	dLn-Pu	0.41	24.76	0.37	24.02	0.40	25.65	0.32	18.88	0.59	30.39	0.52	26.85
	n*Pc-Pu	0.30	3.29	0.26	2.86	0.28	3.12	0.24	2.60	0.43	4.56	0.98	4.00
	n*Ln-Pu	0.47	13.41	0.42	12.07	0.50	13.79	0.54	14.90	0.69	9.27	0.37	13.07

*NP* Neutral Position; *LDT* Lateral Distraction Test

### Intra-Rater Reliability for 3D-LDT

Intra-rater reliability (ICC) for measurements taken from 3D surface images was greater than 0.95 for five of the six measurements: equal to 0.95 for distance from nasal limbus to punctum in LDT (Table 4).

The MAD were less than 1 mm for all the six measurements. Two measurements (distance from pupil center to punctum both in NP and LDT) of the six measurements had a REM between 1 and 4%, two measurements (distance from limbus to punctum in NP and distraction distance from pupil center to lower punctum in LDT) had a REM between 4 and 7%. Two measurements (normal distance from limbus to punctum in LDT and distraction distance from nasal limbus to lower punctum in LDT) had a REM over 10%. The TEM was less than 1 mm (< 1 unit) for all the six measurements. Two measurements (distance from pupil center to punctum both in NP and LDT) had a %TEM was between 1 and 4%. Measurement of distance from pupil center to lower punctum in LDT had a %TEM between 4 and 7% and the measurement from limbus nasal center to punctum in NP had a %TEM between 7 and 10%. The %TEM for normal distance from limbus to punctum in distraction position and the distraction distance from nasal limbus to lower punctum were over 10% (Table 5).

### Inter-Rater Reliability for 3D-LDT

Inter-class correlation (ICC) for 3D image measurements were greater than or equal to 0.95 for 5 of the 6 measurements, between 0.90 and 0.94 for the distance from nasal limbus to punctum in the LDT (Table 4).

MAD were less than 1 mm for all the 6 measurements. Two measurements (distance from pupil center to punctum in both NP and LDT) of the six measurements had a REM between 1 and 4%, two measurements (distance from limbus to punctum in NP and distraction distance from pupil center to lower punctum) had a REM between 7 and 10%. The REM for normal distance from limbus to punctum in LDT and the distraction distance from nasal limbus to lower punctum were over 10%.

The TEM was less than 1 mm for all the six measurements. Two measurements (distance from pupil center to punctum both in the NP and LDT) of the six measurements had a %TEM between 1 and 4%. The measurements of distance from limbus to punctum in NP, and the measurement of distraction distance from pupil center to lower punctum LDT were between 4 and 10%. The %TEM for normal distance from limbus to punctum in LDT and the distraction distance from nasal limbus to lower punctum were over 10% (Table 5).

### Inter-Method Reliability for 3D-LDT

Intraclass correlation (ICC) comparing observer one and observer two for all participants were greater than 0.90 for distance from pupil center to punctum in NP; between 0.80 and 0.89 for distance from limbus to punctum in rest position; and between 0.70 and 0.80 for the normal distance from pupil center and nasal limbus to punctum in LDT, and distraction distance from pupil center to punctum in LDT, and less than 0.70 for the distraction distance from nasal limbus to punctum in LDT (Table 4).

MAD were less than 1 mm for all the 6 measurements. The measurement of the distance from pupil center to punctum in NP had a REM between 1 and 4%. Two measurements (distance from nasal limbus to punctum in NP and LDT) had a REM between 4 and 7%. The REM for normal distance from limbus to punctum in LDT was between 7 and 10%. The distraction distance from pupil center to lower punctum was 12.04%, and the REM for distraction distance from nasal limbus to lower punctum was over 30%. The TEM was less than 1 mm for five of the six measurements. One measurement for the distraction distance from pupil center to punctum in LDT had a TEM of 1.11 mm. Two measurements (distance from pupil center to punctum both in NP and LDT) of the six measurements had a %TEM between 1 and 4%. The measurement of distance from limbus to punctum in NP had a %TEM of between 4 and 7%. Both the %TEM for distraction distance from pupil center and normal distance from nasal limbus to punctum in LDT were between 10 and 20%. The %TEM for distraction distance from nasal limbus to lower punctum in LDT were over 20%. (Table 5).

## Discussion

All the anthropometric measurements for the punctum position taken on 3D images by means of the VECTRA^M3^ System in NP demonstrated high reliability (Table 5), consistent with the previous studies using this system in our research group for the periocular anthropometry [13, 27, 28]. All the measurements in the LDT observed by this indirect way of VECTRA^M3^ System also demonstrated high reliability, despite the relatively poor result for the distance from limbus nasal center to the lower punctum (Table 4). Digital image processing techniques have been successfully used to examine the palpebral contour in different pathologies of the eyelid position [33]. Additionally, Daniella de Paiva Almeida Stuchi et al, investigated the intra- and inter-observer reliability of a modified distraction test based on the 3D imaging technique for assessing the horizontal tension of lower eyelid [34]. However, no studies used digital images to analyze the medial canthal tension laxity so far. Moreover, previous studies on the MCT laxity were only qualitatively graded [8, 35, 36]. To the best of our knowledge, this is the first study investigating the reliability of using 3D image for analyzing the MCT laxity. Furthermore, our study firstly demonstrated the mean NP and distraction position of the lower punctum in a quantitative way in a normal population.

Our study showed that almost all measurements originated from the NP had higher reliability (MAD, REM, TEM, %TEM and ICC) than those originated from the LDT (Table 5), particularly within the intra-observer and inter- method categories. Clearly, the 3D images taken in the NP avoid the bias that may be caused by the image torsion when performing the LDT. Images taken in NP provided the overall better precision than in the distraction test. These results suggest that the VECTRA^M3^ System is capable of excellent reliability for the evaluation the punctum position for the normal population or patients with lower eyelids diseases. More specifically, the results could be introduced to further evaluation for lower eyelid diseases such as ectropion especially for patients with medial ectropion or symptom of epiphora, and so on.

For all the digital images, we set two different distance (Pc-Pu and Ln-Pu) according to two reference vertical lines, with one across the pupil center (Pc) and another across the corneal limbus nasal center (Ln). Previous studies [8, 11, 37] normally used the vertical line across the corneal limbus nasal center to define the resting position or the distraction distance of the lower punctum in the direct measurements. However, in the indirect way of using 3D images [13, 27, 28, 34], pupil center was the basement for almost all the landmarks. Hence, we developed a reliability comparison between the two reference vertical lines. In our study, measurements for both vertical lines demonstrated high score of ICC in intra- rater, inter-rater and inter-method, despite the significant difference of nPc-Pu for inter-methods reliability and n*Pc-Pu for intra-rater reliability, which were not clinically significant (≤ 1 mm) for all of them (Table 5). Additionally, no significant difference was found between them in NP for MAD, REM, TEM and %TEM in intra- rater, inter-rater and inter-method reliability (Table 4). Admittedly, the score of inter-methods reliability for n*Pc-Pu and dPc-Pu´ was relatively higher than those of intra- and inter-rater reliability, despite individual observers having trained together. Nevertheless, in the distraction test, the overall reliability of the distance according to limbus nasal center was lower than that of pupil center, which indicated that the distraction distance calculated from the pupil center was more reliable and repeatable for the evaluation of the MCT laxity than that from the limbus nasal center to the lower punctum (Table 4).

Furthermore, our study demonstrated the possibilities in the application for different pathological conditions. Evaluation of lower eyelid malpositions plays a vital role in the diagnosing of eyelid disease as well as the designation of surgeries, and the follow-up project is also essential for the evaluation of treatment effects. However, the main evaluation of lower eyelid malpositions, especially the medial canthal malposition is not quantitative due to the limited method [38]. In our study, with the application of the 3D stereophotography and our landmarks system, the pre-operative malposition could be evaluated quantitatively (Fig. 3), and the post-operative follow-up could be recorded for quantitative observation and comparison (Fig. 4). Additionally, the findings of the present study build the essential basis to introduce this novel 3D-LDT in the clinical routine and to conduct follow-up projects investigating variations of repair techniques and their failure rate.

The possible limitation of this study might be the challenge to keep the participants looking into the same position when performing the LDT, especially for the sensitive ones. For optimal image, an individual being captured must keep a consistent head position and visual focus direction. To minimize errors caused by the processing of LDT, repeated captures and cooperative with another operator are necessary.

In conclusion, the high intra-rater, inter-rater and inter-method reliability found in this study was due to the easy application of the technique and analysis of the results. Our novel standardized three-dimensional lateral distraction test (3D-LDT) seems to be a feasible and highly reliable method to assess medial canthal tendon (MCT) laxity. Furthermore, pathological eyelid conditions were not included in this study for the identification error of all the landmarks. Therefore, follow-up studies are necessary to investigate the reliability of this 3D-LDT on pathological cases as well as to evaluate the correlation of these measurements with subjective patients’ complaints and various disease severity of eyelid abnormalities. Lastly, to our best knowledge, this is the first study quantifying the reliability of the 3D-LDT for evaluating the MCT laxity based on digital image processing.

## Data Availability

All authors have full control of all primary data and they agree to allow to review their data upon request.
